# Identification of exercise‐regulated genes in mice exposed to cigarette smoke

**DOI:** 10.14814/phy2.15505

**Published:** 2022-11-02

**Authors:** Lars Aakerøy, Chew W. Cheng, Pavla Sustova, Nathan R. Scrimgeour, Sissel Gyrid Freim Wahl, Sigurd Steinshamn, T. Scott Bowen, Eivind Brønstad

**Affiliations:** ^1^ Department of Thoracic Medicine St. Olavs Hospital, Trondheim University Hospital Trondheim Norway; ^2^ Department of Circulation and Medical Imaging, Faculty of Medicine and Health Science Norwegian University of Science and Technology Trondheim Norway; ^3^ Leeds Institute of Cardiovascular and Metabolic Medicine University of Leeds Leeds UK; ^4^ Department of Pathology St. Olav Hospital, Trondheim University Hospital Trondheim Norway; ^5^ School of Biomedical Sciences, Faculty of Biological Sciences University of Leeds Leeds UK

**Keywords:** Cigarette smoke, endothelial, high‐intensity interval training, transcriptome

## Abstract

Cigarette smoke (CS) is the major risk factor for COPD and is linked to cardiopulmonary dysfunction. Exercise training as part of pulmonary rehabilitation is recommended for all COPD patients. It has several physiological benefits, but the mechanisms involved remain poorly defined. Here, we employed transcriptomic profiling and examined lung endothelium to investigate novel interactions between exercise and CS on cardiopulmonary alterations. Mice were exposed to 20 weeks of CS, CS + 6 weeks of high‐intensity interval training on a treadmill, or control. Lung and cardiac (left and right ventricle) tissue were harvested and RNA‐sequencing was performed and validated with RT‐qPCR. Immunohistochemistry assessed pulmonary arteriolar changes. Transcriptome analysis between groups revealed 37 significantly regulated genes in the lung, 21 genes in the left ventricle, and 43 genes in the right ventricle (likelihood‐ratio test). Validated genes that showed interaction between exercise and CS included angiotensinogen (*p* = 0.002) and resistin‐like alpha (*p* = 0.019) in left ventricle, with prostacyclin synthetase different in pulmonary arterioles (*p* = 0.004). Transcriptomic profiling revealed changes in pulmonary and cardiac tissue following exposure to CS, with exercise training exerting rescue effects. Exercise‐regulated genes included angiotensinogen and resistin‐like alpha, however, it remains unclear if these represent potential candidate genes or biomarkers that could play a role during pulmonary rehabilitation.

## Graphical abstract

In an experimental murine model exposed to cigarette smoke and high‐intensity interval training we performed RNA‐sequencing, RT‐qPCR and immunohistochemistry to investigate interactions between exercise and cigarette smoke on cardiopulmonary alterations. We report novel exercise‐regulated genes in smoke‐exposed mice; angiotensinogen and resistin‐like alpha.

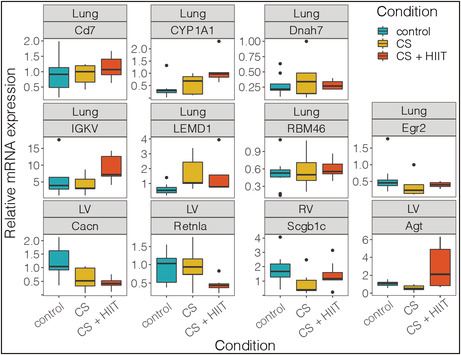

## 1 INTRODUCTION

Cigarette smoke (CS) is a leading cause of morbidity and mortality related to cardiovascular and pulmonary diseases and is considered the number one cause of preventable deaths (The Health Consequences of Smoking‐50 Years of Progress: A Report of the Surgeon General, 2014). It is the major risk factor for COPD, but can induce extrapulmonary effects, for instance to skeletal (limb and respiratory) muscles and the vasculature, that often precede overt pulmonary disease (Elbehairy et al., 2016; Gordon et al., 2011; Seimetz et al., 2011; Darabseh et al., 2021). Physical inactivity has been shown to exacerbate the extrapulmonary impairments associated with prolonged CS in mice (Cielen et al., 2016) while in patients with COPD exercise capacity is considered a better predictor of mortality than lung function (Oga et al., 2003). Whilst current pharmacological therapy for COPD is directed towards symptoms of airflow obstruction and prevention of exacerbations, non‐pharmacological treatment such as exercise training may reverse early cardiovascular manifestations. Evidence from pulmonary rehabilitation support extrapulmonary manifestations in lung disease as treatable traits (Burtin & Wadell, 2021). Using an experimental model for CS exposure and high‐intensity interval training (HIIT) in mice, we reported that HIIT attenuated several cardiopulmonary effects including right ventricular (RV), respiratory muscle and endothelial impairments that occured before onset of overt emphysema (Bowen et al., 2017). The involved mechanisms, however, remain poorly defined.

Genetic mutations confer susceptibility to COPD. The archetype is alpha‐1‐antitrypsin (A1AT) deficiency, but more complex interactions between specific gene loci and environmental factors have been suggested (Cho et al., 2014). CS might affect DNA transcription by epigenetic alterations or by directly damaging DNA by smoke components (Kopa & Pawliczak, 2018). Past RNA‐sequencing studies in mice and humans have confirmed that both CS per se and COPD affect the transcriptome in blood and lung tissue (Huan et al., 2016; Jeong et al., 2018; Vishweswaraiah et al., 2018). However, it remains uncertain whether such genetic alterations are directly responsible for the development of COPD including, in particular, any of its cardiovascular manifestations. Furthermore, it is unknown if exercise may prove beneficial by remodeling the transcriptome landscape. An alternative linking mechanism may include a role for CS‐induced endothelial dysfunction (Polverino et al., 2018). Previous studies have shown that CS affects the expression of enzymes catalyzing the formation of important vasoactive mediators in the pulmonary vasculature (Seimetz et al., 2011; Nana‐Sinkam et al., 2007). The effects of HIIT on these CS‐induced changes are not established.

In line with this, the current study aimed to identify possible endothelial and transcriptomic changes in lung and cardiac tissue by interrogating the interaction between exercise and CS exposure using unbiased RNA‐sequencing in an experimental murine model.

## 2 METHODS

### 2.1 Animals, smoke exposure and exercise protocol

Details about the animals, protocols for CS exposure and tissue preparation have been described extensively elsewhere (Bowen et al., 2017). The study was approved by the Norwegian Animal Research Authority (FOTS case number 5855). Briefly, 10 week old A/JOlaHSD female mice (Harlan Laboratories) were randomly divided into three groups and exposed to room air (*n* = 10), CS (*n* = 10) or CS plus HIIT (*n* = 11). CS exposure (Research Cigarettes 3R4F, University of Kentucky) was performed for 6 hours per day, 5 days per week for 20 weeks in an exposure chamber (TE‐10, Teague Enterprises). The exercise intervention has been outlined in full detail previously (Bowen et al., 2017) In brief, it commenced after CS exposure was completed and consisted of HIIT being performed five times per week for 6 weeks on a treadmill (Exer‐3/6, Colombus Instruments) with 25° incline. Each session comprised 10 intervals (4 minutes at 85%–90% peak work rate) separated by a 2‐minute recovery (50%–60% peak work rate). Exercise capacity was assessed in all mice by measuring peak oxygen uptake via a closed metabolic chamber. As the training programme progressed, running speed during intervals could be gradually increased up to 18 m/min. At sacrifice, LV, RV and left lung were dissected, and immediately frozen in liquid nitrogen and stored at −80 for subsequent profiling.

### 2.2 RNA‐sequencing

Total RNA was extracted from 91 (RV (*n* = 29), LV (*n* = 31) and lung (*n* = 31)) mouse tissue samples, using RNeasy® Fibrous Tissue Mini Kit (Qiagen Sciences Inc.) and RNeasy Mini Kit (Qiagen) according to manufacturer's instructions. RNA quantity, quality/integrity and purity were evaluated using Qubit (Thermo Fisher Scientific), Bioanalyzer (Agilent Technologies) and NanoDrop (Thermo Fisher).

According to manufacturer's protocol, RNA sequencing libraries were generated using the SENSE mRNA‐Seq library prep kit (Lexogen GmbH). In brief, 500 ng of total RNA was prepared and incubated with magnetic beads coated with oligo‐dT. RNAs other than mRNA were removed by washing. Library preparation was then initiated by random hybridization of starter/stopper heterodimers to the poly(A) RNA still bound to the magnetic beads. These starter/stopper heterodimers contain Illumina‐compatible linker sequences. A single‐tube reverse transcription and ligation reaction extended the starter to the next hybridized heterodimer, where the newly‐synthesized cDNA insert was ligated to the stopper. Second‐strand synthesis was performed to release the library from the beads. After adding the adaptors and indexes, the resulting double‐stranded library was purified and amplified (14 PCR cycles). Finally, qPCR quantitated libraries using KAPA Library Quantification Kit (Kapa Biosystems Inc.) and validated using Agilent High Sensitivity DNA Kit on a Bioanalyzer (Agilent). The DNA fragments size range was 200–450 bp with an average library size of 245 bp.

Before sequencing, the libraries were quantified (KAPA Library Quantification Kit [Illumina/ABI Prism]), normalized and pooled. Quantitated libraries were further diluted to 2.4 nM and subjected to clustering by a cBot Cluster Generation System on HiSeq4000 flow cells (Illumina Inc.) according to manufacturer's protocol. Finally, single‐end read sequencing was performed for 75 cycles on HiSeq4000 according to manufacturer's instructions (Illumina). FASTQ files were created with bcl2fastq 2.17 (Illumina) and assessed using the FastQC tool v0.118 (Andrews, 2010) to determine the quality of the raw sequences. Subsequently, the raw sequences were trimmed using TrimGalore v0.6.2 (Krueger, 2015) to remove low‐quality sequences and sequencing adapters. The cleaned reads were mapped to the mouse reference genome (GRCm39) using STAR aligner v2.7 (Dobin et al., 2013). Post‐alignment quality control was performed on the Binary Alignment Map (BAM) files. FeatureCounts v1.6.5 (Liao et al., 2013) was used to derive read counts of each gene, subsequently utilized for differential expression analysis using DESeq2 v1.26 (Love et al., 2014), a Bioconductor package implemented in an R environment. DESeq2 normalizes the raw read counts based on the median of ratios assumptions as previously described (Love et al., 2014). Differential expression analyses were performed based on pairwise comparison, and the genes that achieved a false discovery rate (FDR) threshold <0.05 were considered statistically significant. The likelihood ratio test (LRT) is implemented in DESeq2 and identifies any genes that change expression across the different conditions. Three comparison analyses were performed for each tissue i.e., control versus CS, control versus HIIT and CS versus HIIT.

### 2.3 Quantitative reverse transcription‐polymerase chain reaction

Real‐time polymerase chain reaction (RT‐PCR) validated differentially expressed genes derived from RNA‐sequencing. We selected the genes for validation based on the criteria that the expression levels appeared to be affected by CS but normalized by HIIT. RNA was reverse transcribed to cDNA using QuantiTect Reverse Transcription Kit (Qiagen). PCR reactions were prepared using QuantiNova LNA PCR Assays shown in Table 1 and QuantiTect SYBR Green PCR Kit (Qiagen) according to the manufacturer's protocol. Reactions were cycled using a CFX Opus Real‐Time PCR system (Bio‐Rad Laboratories) and CFX Maestro software (Bio‐Rad). The thermal cycling protocol was as follows: Polymerase activation at 95°C for 2 min, followed by 45 cycles of denaturation at 95°C for 5 seconds, and extension at 60°C for 10 seconds. Data were analyzed using the ΔΔCT method (Livak & Schmittgen, 2001). Rpl, Hprt, and Ppia were used as housekeeping genes for relative quantification.

**TABLE 1:** List of Qiagen probes for targeted genes

Qiagen Geneglobe ID	Target
SBM1225376	Rpl
SBM1225379	Hprt
SBM1225375	Ppia
SBM0849981	Rbm46
SBM0675220	Cd7
SBM0687230	Cyp1a1
SBM0878433	Dnah7b
SBM0767339	Egr2
SBM1058401	Igkv12‐89
SBM0694737	Lemd1
SBM0838798	Agt
SBM1002933	Cacng6
SBM0829928	Retnla
SBM0935597	Scgb1c1

### 2.4 Immunohistochemistry

Formalin‐fixed, paraffin‐embedded (FFPE) tissue blocks were prepared from lung tissue fixed in 10% buffered neutral formalin. Paraffin sections of 4 μm were placed on Thermo Scientific™SuperFrostPlus slides (Thermo Fisher), dried at 37°C overnight, and then baked at 60°C for 60 min prior to staining. The paraffin slides were deparaffinized and pre‐treated for heat‐induced antigen retrieval with a pre‐programmed PT‐Link Instrument (Dako, Agilent) by using Target Retrieval Solution, Citrate pH 6 (Agilent) for 20 min at 97°C, then cooled to 65°C. Endogenous peroxidase activity was quenched by applying Agilent DAKO S2023 peroxidase blocking solution for 10 min. The paraffin slides were incubated with the primary antibody and diluted in Dako REAL Antibody Diluent S2022. After primary antibody incubation, the slides were incubated for 30 min with the DakoEnvision™ K4003 Peroxidase/DAB+ anti‐rabbit. The slides were then incubated with Diaminobenzidine chromogen for 10 min, followed by application of hematoxylin for 30 seconds. Two washing steps (washing buffer + Tween20) were performed between each step of the procedure. The staining procedure was performed with Dako Autostainer Plus.

The following antibodies, concentrations, dilution and incubation conditions were used in this study: Agilent DAKO Von Willebrand factor (A0082, Agilent Dako, 1:400 dilution, 40 min at room temperature), Alpha smooth muscle actin (ab 5694, abcam, 1:400 dilution, 40 min at room temperature), PTGIS/PGIS (ab23668, abcam, 1:300 dilution, 40 min at room temperature), Endothelin (ab117757, abcam, 1:500 dilution, 40 min at room temperature) and anti‐iNOS rabbit antibody (clone SP126, Sigma Aldrich Cat. No. SAB5500152, 1:50 dilution, overnight incubation at 4°C). For staining quality control, we used rabbit IgG isotype control (NBP2‐24891, NOVUS) at the same concentrations and dilutions as respective antibodies.

The slides were evaluated and scored independently by two pathologists (PS and SGFW), blinded to CS and exercise interventions. Positive staining for von Willebrand factor was used for identification of pulmonary vessels, while the thickness of vessel walls was assessed in sections stained with smooth muscle actin. Vessel wall thickness was measured in μm in pulmonary arterioles; larger arteries and veins were excluded. For endothelin 1 (ET1), staining of endothelial cells was graded as either present or absent. The number of prostacyclin synthetase (PTGIS) positive and negative endothelial cells in each section were counted and the results presented as a ratio of positive to negative cells.

### 2.5 Statistical analysis

Descriptive data are presented in the text as mean ± standard deviation. We used one‐way analyses of variance (ANOVA) with Bonferroni post hoc analysis for comparison of continuous variables. Chi‐Square test for independence was used to compare the expression of ET1 between groups. We used *p* < 0.05 as a threshold for indicating statistical significance. Analyses were performed using IBM SPSS Statistics for Windows, version 26.0 (IBM corp.). DESeq2 analysis was carried out in R for Mac, version 4.1.2.

## 3 RESULTS

### 3.1 Transcriptomic profiling

**FIGURE 1:**
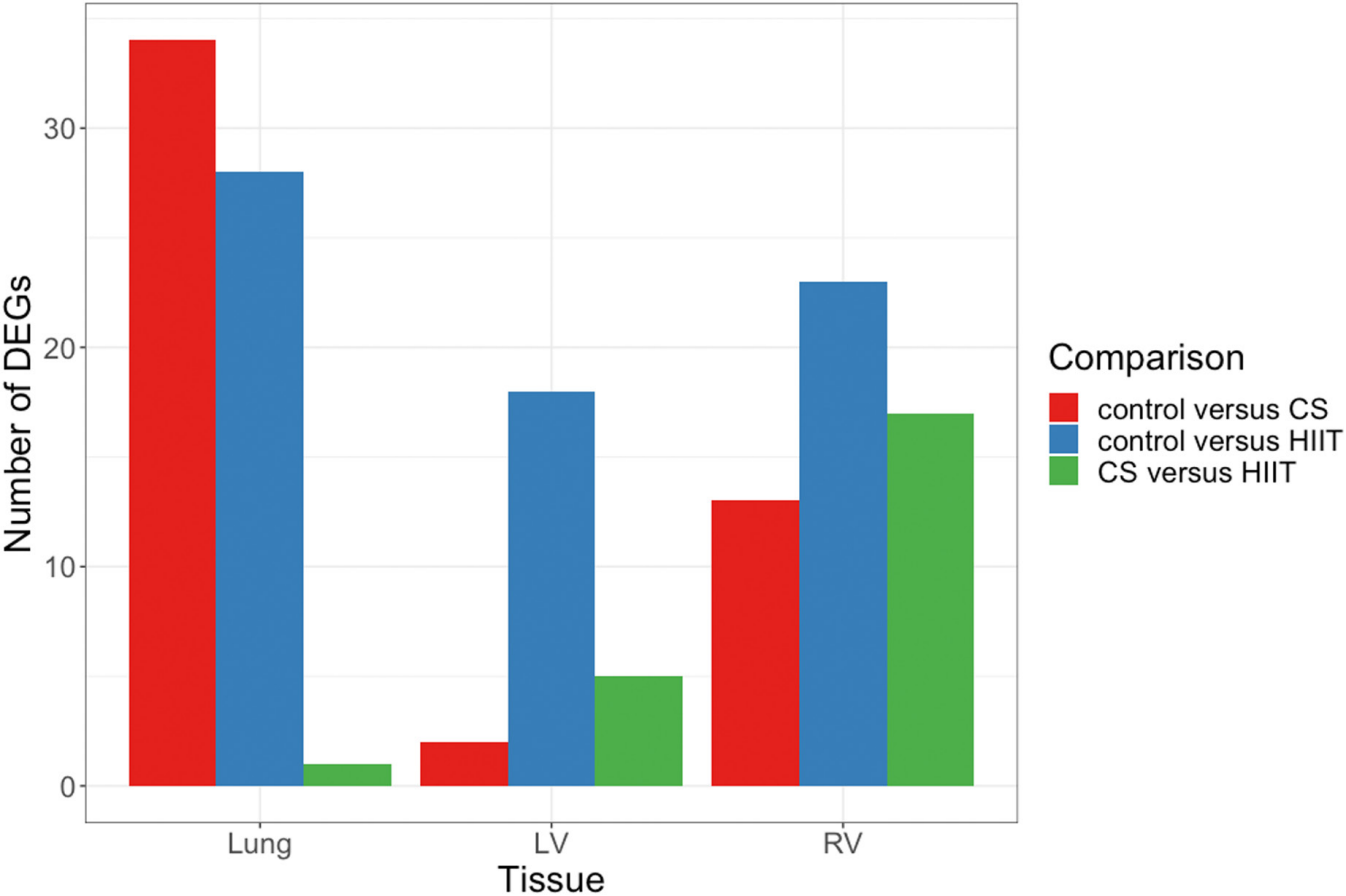
Differentially expressed genes identified in different tissues according to pairwise comparison. Statistically significant genes are identified based on adjusted *p*–value <0.05. CS, cigarette smoke; HIIT, High‐intensity interval training; LV, left ventricle; RV, right ventricle.

We first performed full RNA‐sequencing in the lung, RV and LV for unbiased identification of potential candidate genes to assess the interaction between CS and exercise training. Using pairwise comparison analyses, 34, 28, and 1 differentially expressed genes from the lung were detected in control versus CS, control versus HIIT and CS versus HIIT respectively (Figure 1 and Data [Link], [Link] [https://figshare.com/s/65fec9f16de0f54430ef]). Additionally, 2, 18, and 5 differentially regulated genes were identified in the LV while 13, 23, and 17 genes were detected in the RV across three comparisons (Data [Link], [Link] [https://figshare.com/s/65fec9f16de0f54430ef]).

**FIGURE 2:**
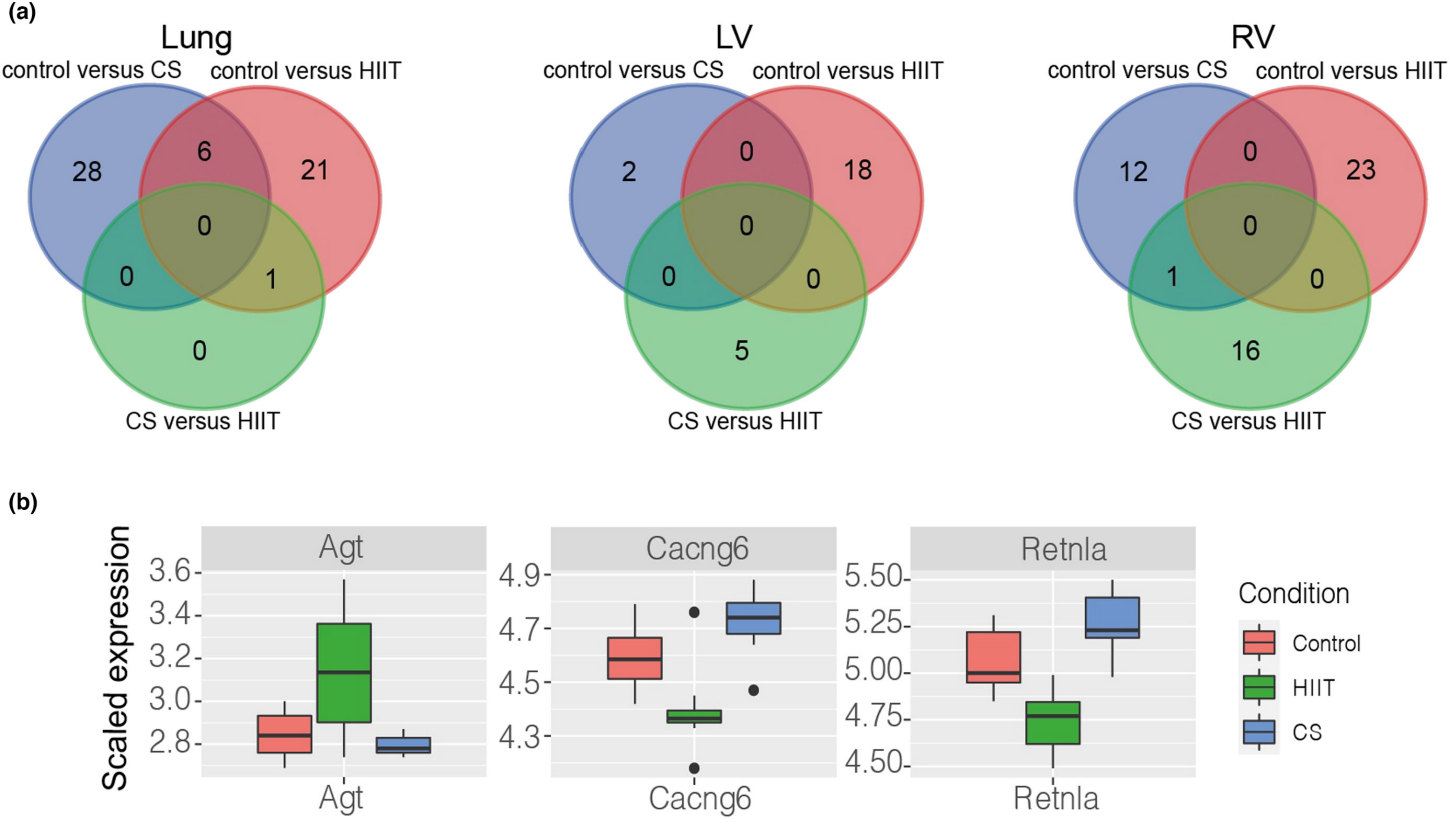
(a) Venn diagrams of differentially expressed genes identified in different comparisons and tissues. (b) Likelihood ratio test performed in the left ventricle cohort. The boxplots represent the scaled expression in each condition and are colored according to conditions (red: control, green: HIIT and blue: smoke exposure). The expression profile of the selected genes in HIIT are either increased or decreased significantly compared to control and smoke exposure. CS, cigarette smoke; HIIT, High‐intensity interval training; LV, left ventricle; RV, right ventricle.

Following pairwise comparison analyses, we used LRT to identify genes that were significantly differentially expressed across three conditions. Based on LRT, 37, 21 and 43 genes were found differentially regulated in the lung, LV and RV across three conditions (Data [Link], [Link] [https://figshare.com/s/65fec9f16de0f54430ef]). We focused on the specific pattern within the conditions, that is, genes that exhibited high expression in CS exposure while HIIT showed lower expression than control and CS exposure, or vice versa (Figure 2 and Figures [Link], [Link] [https://figshare.com/s/65fec9f16de0f54430ef]).

### 3.2 Validation of potential mechanisms

**FIGURE 3:**
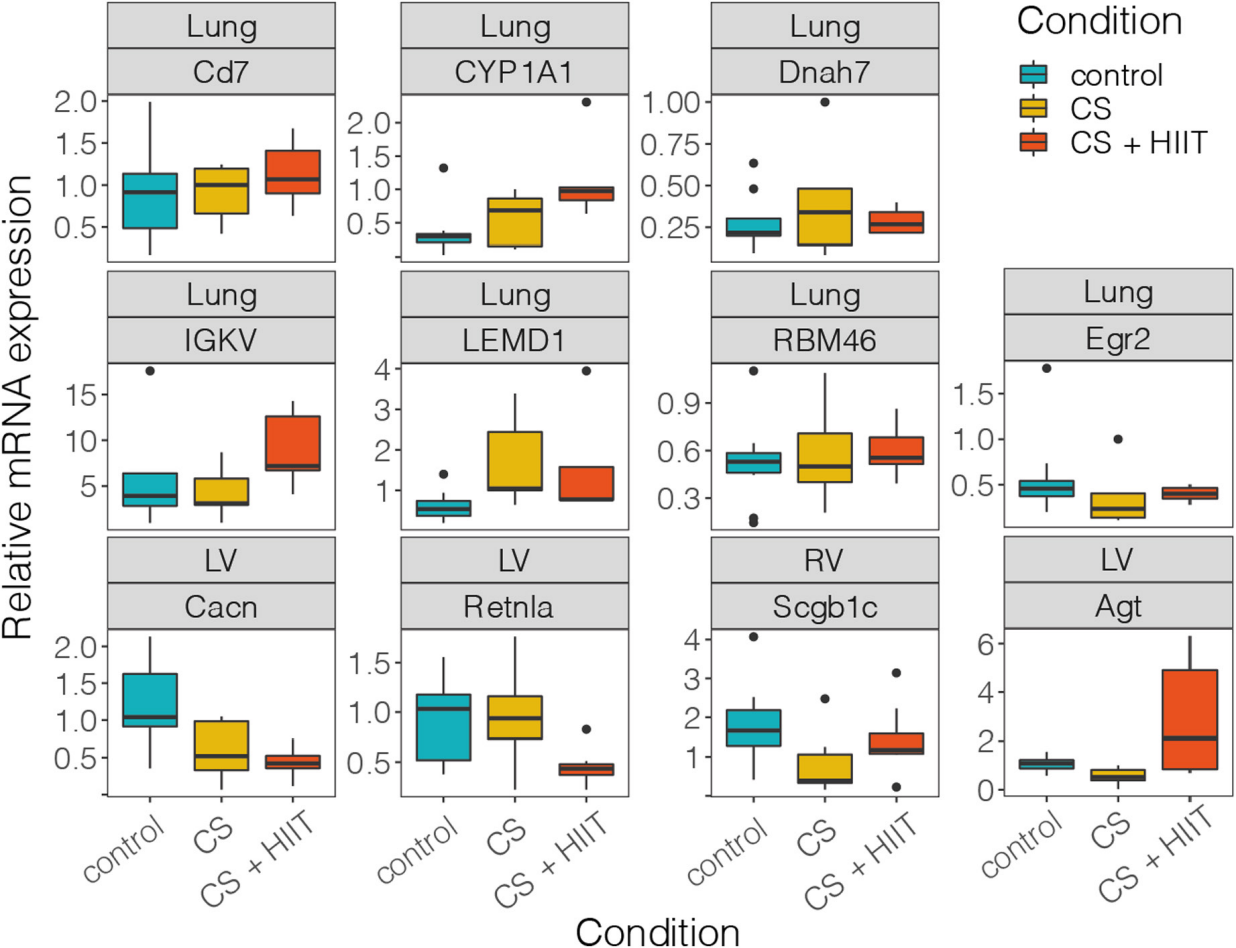
RT‐qPCR of selected genes from various tissues. Boxplot colored according to condition. CS, cigarette smoke; HIIT, High‐intensity interval training; LV, left ventricle; RV, right ventricle.

Findings of gene expression were confirmed via RT‐PCR to follow the same pattern as RNA sequencing data. The most notable findings were from LV and included angiotensinogen (Agt) and resistin‐like alpha (Retnla), which were significantly regulated (*p* = 0.002 and *p* = 0.019, respectively) (Figure 3). Bonferroni's test to identify between group differences demonstrated that the influence of gene expression was due to HIIT, which significantly upregulated Agt compared to controls (*p* = 0.023) and CS (*p* = 0.002) and downregulated Retnla compared to controls (*p* = 0.046) and CS (*p* = 0.039). In contrast, the comparison between controls and CS‐exposed animals was unchanged (*p* = 1.0) for both these gene targets. Exceptions to the gene expression pattern included Cagng6, Cyp1A1 and Rbm46, where CS + HIIT expression mean values deviated further from the controls than CS itself.

Immunohistochemistry analysis further confirmed integrity of vessel structure and function. Vessel wall thickness was measured from several pulmonary arterioles (*n* = 14–41) from each animal, yielding a total of 237–272 separate measurements from each group, but no differences were found between control, CS and CS + HIIT (3.06 ± 1.02, 3.37 ± 1.27 and 2.91 ± 1.14 μm, respectively; *p* = 0.413). Similarly, there was no difference in the staining for ET1 between controls, CS and CS + HIIT (present vs. absent staining was 2 vs. 8, 3 vs. 7 and 5 vs. 5, respectively; *p* = 0.350), whereas inducible nitric oxide synthetase (iNOS) was not detected at all. In contrast, we observed a difference in the ratio of endothelial cells that stained with PTGIS between controls, CS and CS + HIIT (1.91 ± 0.91, 0.93 ± 0.76 and 0.70 ± 0.43, respectively; *p* = 0,004) with between group differences revealing difference between controls and CS (*p* = 0.02) and controls and CS + HIIT (*p* = 0.006) but without any exercise effect (*p* = 1.0).

## 4 DISCUSSION

This study aimed to identify potential candidate genes to explain how exercise training protects against CS‐induced cardiopulmonary alterations. Overall our transcriptome analysis identified two exercise‐regulated genes (Agt and Retnla) in mice exposed to CS. HIIT did not influence some main markers of pulmonary vascular function.

### 4.1 Transcriptome in response to CS

We suggest for the first time two genetic markers in LV (Agt and Retnla) that could be involved in the cardiopulmonary interplay after exposure to CS and HIIT. Interestingly, in the same mice we have previously reported that the exercise intervention significantly improved both peak oxygen uptake as well as LV function, as measured by LV ejection fraction and LV fractional shortening (Bowen et al., 2017). A possible link between the genetic markers Agt and Retnla, improved cardiac function and exercise capacity could therefore be hypothesized. Although speculative, this hypothesis could be extended to involve prognosis since exercise capacity is considered an important predictor of mortality in COPD (Oga et al., 2003).

Agt is a member of the non‐inhibitory serpin (serine protease inhibitor) superfamily, along with A1AT. It is the unique precursor of the renin‐angiotensin system (RAS), whose 10 N‐terminal amino acids are cleaved by renin in order to form angiotensin I. The importance of RAS in maintaining normal cardiovascular performance, for instance by regulating blood pressure and the homeostasis of sodium and water, is well‐known. Furthermore, many of its constituents are important targets for pharmacological therapy in various cardiovascular diseases. As such, the contribution of Agt has received less attention than the constituents of RAS further downstream. Still, it has recently been implicated in hypertension, atherosclerosis, myocardial hypertrophy, heart failure and atrial fibrillation (Xu et al., 2021). Certain Agt gene polymorphisms have been reported to increase the risk of cardiovascular disease in humans (Li et al., 2021) and others related to cardiac hypertrophy (Wang et al., 2014). There is thus growing evidence that Agt is more than a mere passive substrate for RAS. So far, there is limited data regarding the effects of exercise on this gene, in particular after exposure to CS. A previous study in rats found reduced gene expression of Agt after high versus low intensity exercise (Tucker et al., 2015). This contrasts with our findings of an upregulated Agt after HIIT but may be explained by the previous study analyzing a homogenized mixture of rat renal and hepatic tissues in the setting of chronic kidney disease (Tucker et al., 2015). The increase in Agt expression in LV observed in our study is novel and may speculatively be implicated in the cardiac hypertrophy observed after exercise and improved LV function in these animals (Bowen et al., 2017).

The Retnla gene in mice encodes resistin‐like molecule alpha (RETNLA or RELMα), also known as found in inflammatory zone 1 (FIZZ1) and hypoxia‐induced mitogenic factor (HIMF). Recently it was reported to be involved in several pathological processes, including angiogenesis, vasoconstriction, inflammation and fibrosis and implicated in pulmonary, cardiovascular and metabolic diseases (Lv & Liu, 2021; Lin & Johns, 2020; Kumar et al., 2018). With regards to the cardiovascular system, HIMF may, for instance, play a critical role in the development of cardiomyocyte hypertrophy and pulmonary hypertension via hypoxia‐induced factor‐1 α (HIF‐1 α) mechanisms (Kumar et al., 2018; Johns et al., 2016) or pulmonary hypertension through phosphatidylinositol 3‐kinase (PI3K) pathways (Teng et al., 2003), and targeting HIMF may represent a potential therapeutic strategy (Kumar et al., 2018). A previous study of rat lung tissue showed that exercise reversed changes to mRNA expression following CS (Ma et al., 2013). The findings in our study are, however, the first to suggest that HIIT downregulates the expression of this gene in the LV of mice exposed to CS.

### 4.2 Pulmonary vascular structure and function

We did not observe transcriptomic changes that clearly related to endothelial function. HIIT also did not change immunohistochemistry expression for vasoactive mediators. Corroborating findings in a previous study (Nana‐Sinkam et al., 2007), we found that CS reduced expression of PTGIS in endothelial cells of pulmonary arterioles but HIIT had no influence. Our data are the first to have assessed the effect of HIIT on this change. Past reports have indicated effects of HIIT on plasma levels of ET1 in humans and rats and cardiac tissue in rats (Izadi et al., 2018; Wang et al., 2020), and shown upregulated iNOS in the pulmonary vasculature in mice exposed to CS (Seimetz et al., 2011). We did not observe any significant difference in the staining for ET1 in endothelial cells of the smaller pulmonary arterioles and did not detect iNOS expression at all in these vessels. Chronic CS has been associated with a significantly diminished decrease in ET1 in response to acute exercise when compared to non‐smokers (Cooke et al., 2015). A similar mechanism could perhaps explain the lack of significance in our data.

### 4.3 Limitations

To the best of our knowledge, this is the first study of transcriptomic profiling in multiple tissues following CS and HIIT, which resulted in identification of two possible exercise‐regulated gene targets. However, while we validated targets via mRNA expression, we did not confirm this on a protein level. The levels of transcriptome and endothelial changes with CS were perhaps small considering the intervention, however CS exposure ceased for all animals at the start of the exercise intervention, that is, for 6 weeks. This is a relatively long time in the life span of a mouse to reverse some CS‐dependent effects. On the other hand, it might serve as a good model for smoking cessation. Secondly, degradation in lung tissue samples in the PCR‐analysis led to a low number of observations and possibly increased risk of type II errors. Changes related to CS could primarily be expected in the lung. Similarly, sample degradation and low sample size may explain why validated targets did not always follow RNA sequencing results. Considering the complex interplay between organs in various pathophysiological mechanisms, it is unlikely that a single gene or endothelial mediator could explain the effects observed in mice exposed to both CS and HIIT. Several markers other than the ones we examined could also have been of interest to study further.

## 5 CONCLUSION

Overall transcriptome analysis identified two exercise‐regulated genes from LV in mice exposed to CS and HIIT. A possible link between Agt and Retnla, improved cardiac function and exercise capacity could be hypothesized, however, it remains unclear if these represent potential candidate genes or biomarkers related to exercise training or during pulmonary rehabilitation.

## AUTHOR CONTRIBUTIONS

LA, SS, TSB and EB contributed to the conception and design. LA, CWC, PS, SGFW, NRS, TSB and EB contributed to the acquisition of data, analysis and interpretation. LA and CWC drafted the article. All authors significantly contributed to and critically revised the manuscript.

## FUNDING INFORMATION

This work was supported by the liaison committee between the Central Norwegian Health Authority (RHA) and the Norwegian University of Science and Technology (NTNU).

## Supporting information


Figure S1

Figure S2

Figure S3

Data S1

Data S2
