# Supplementary figures and images for: Identification of exercise‐regulated genes in mice exposed to cigarette smoke
Source: Physiol Rep. 2022 Nov 2;10(21):e15505. doi: 10.14814/phy2.15505 (PMC9630761; doi:10.14814/phy2.15505)

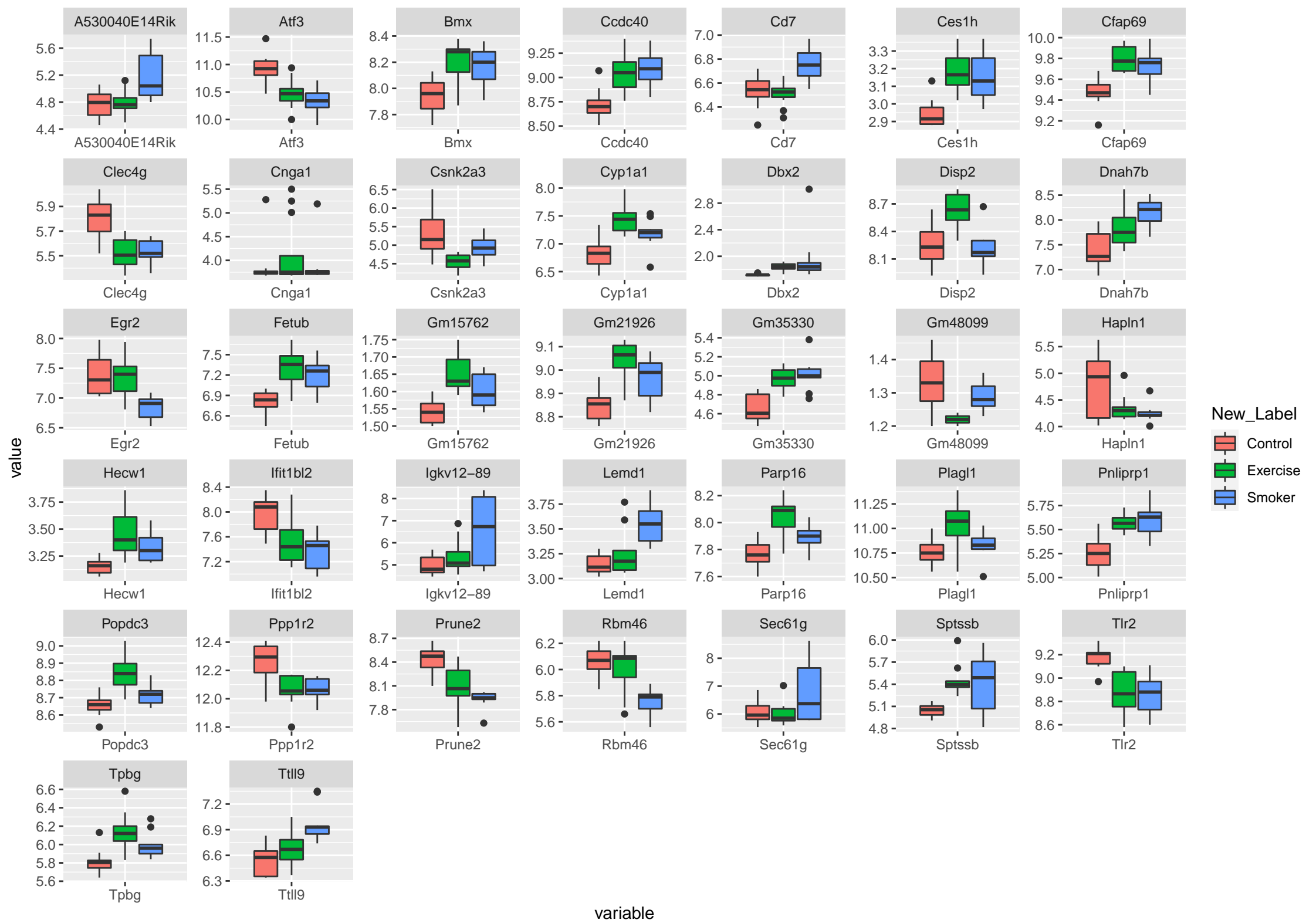

Supplement: Supplementary file 1 — Figure S1 [file PHY2-10-e15505-s002.pdf]

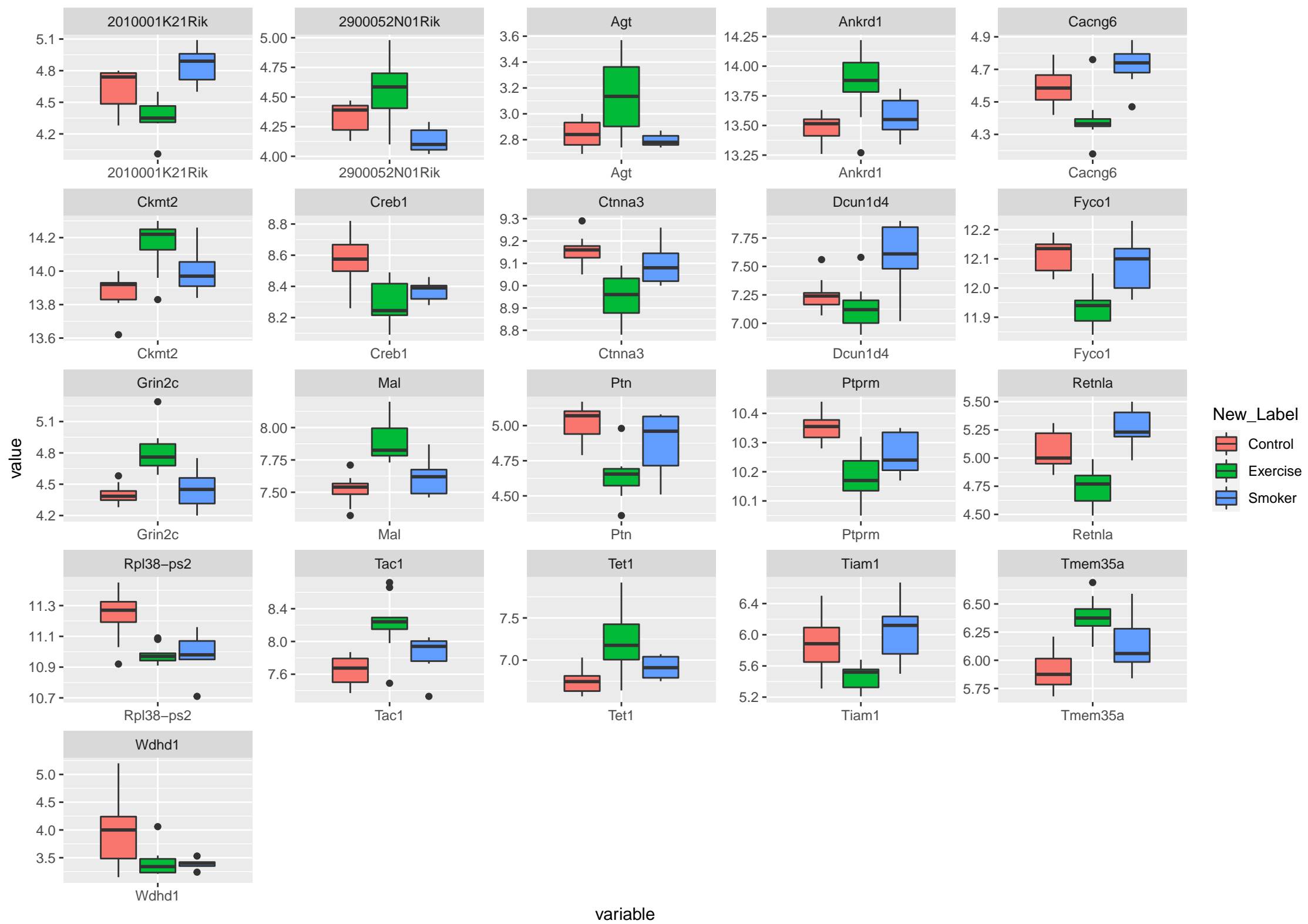

Supplement: Supplementary file 2 — Figure S2 [file PHY2-10-e15505-s005.pdf]

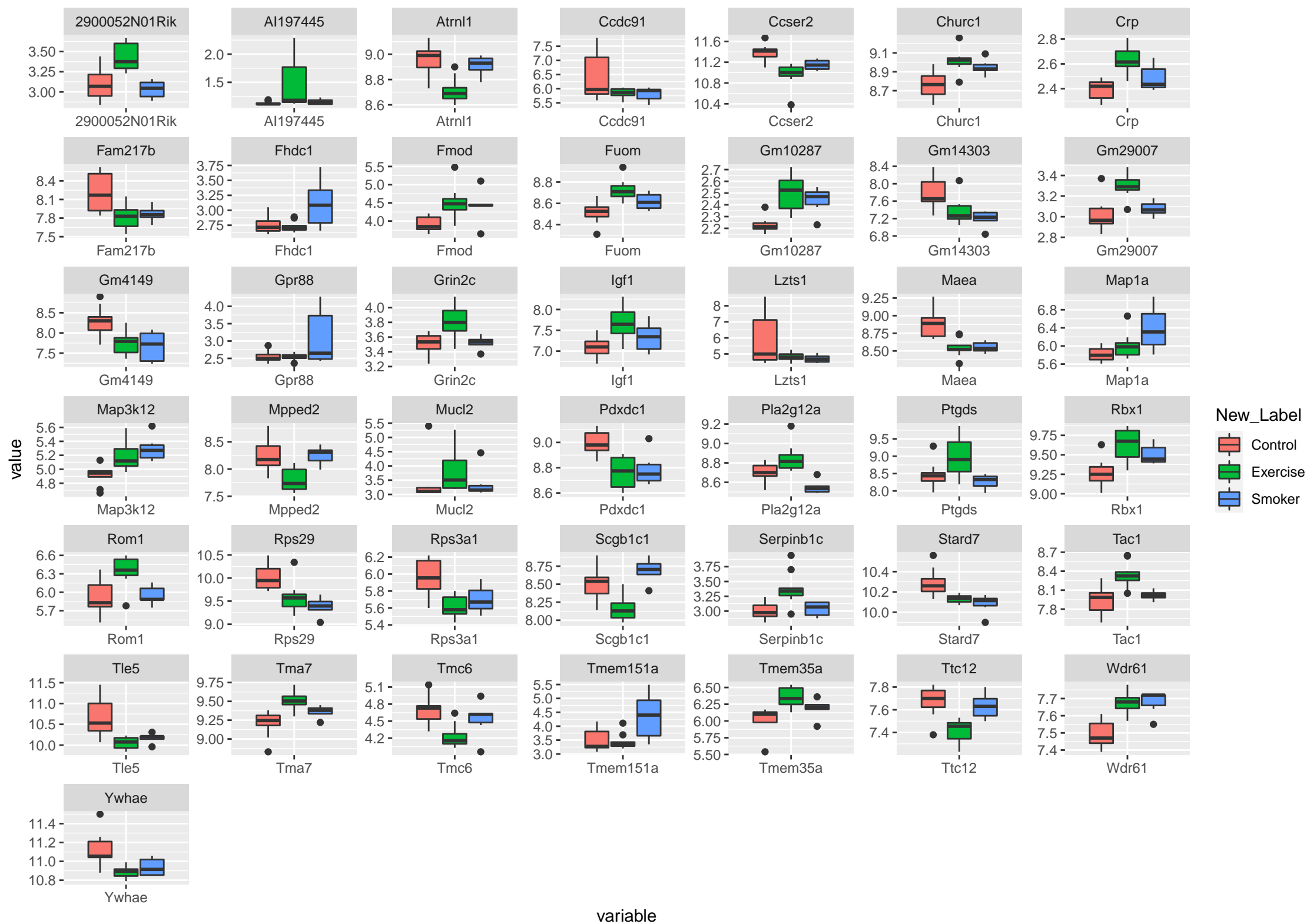

Supplement: Supplementary file 3 — Figure S3 [file PHY2-10-e15505-s004.pdf]
