# Supplementary material for: Identification of exercise‐regulated genes in mice exposed to cigarette smoke
Source: Physiol Rep. 2022 Nov 2;10(21):e15505. doi: 10.14814/phy2.15505 (PMC9630761; doi:10.14814/phy2.15505)
Supplement: Supplementary file 5 — Data S2 [file PHY2-10-e15505-s003.docx]

**Supplementary figure 1.** Differentially expressed genes in lung tissue across three conditions; room air, cigarette smoke and cigarette smoke plus HIIT. The likelihood ratio test was used to identify the genes based on adjusted P-value <0.05. The boxplots represent the scaled expression in each condition and are coloured according to conditions (red: room air, green: cigarette smoke plus HIIT and blue: cigarette smoke).

HIIT = high intensity interval training.

**Supplementary figure 2**. Differentially expressed genes in left ventricle tissue across three conditions; control, cigarette smoke and cigarette smoke plus HIIT. The likelihood ratio test was used to identify the genes based on adjusted P-value <0.05. The boxplots represent the scaled expression in each condition and are coloured according to conditions (red: room air, green: cigarette smoke plus HIIT and blue: cigarette smoke).

HIIT = high intensity interval training.

**Supplementary figure 3**. Differentially expressed genes in right ventricle tissue across three conditions; room air, cigarette smoke and cigarette smoke plus HIIT. The likelihood ratio test was used to identify the genes based on adjusted P-value <0.05. The boxplots represent the scaled expression in each condition and are coloured according to conditions (red: room air, green: cigarette smoke plus HIIT and blue: cigarette smoke).

HIIT = high intensity interval training.
